# Longitudinal evaluation of laboratory results and method precision in worldwide erythropoietin external quality assessments

**DOI:** 10.3389/fmolb.2024.1390079

**Published:** 2024-06-21

**Authors:** Luisa Toll, Nathalie Weiss, Laura Vierbaum, Ingo Schellenberg, Mario Thevis, Folker Wenzel

**Affiliations:** ^1^ Faculty of Medical and Life Sciences, Furtwangen University, Villingen-Schwenningen, Germany; ^2^ Institute of Biochemistry/ Center for Preventive Doping Research, German Sport University Cologne, Cologne, Germany; ^3^ INSTAND e.V., Society for Promoting Quality Assurance in Medical Laboratories, Düsseldorf, Germany; ^4^ Institute of Bioanalytical Sciences (IBAS), Center of Life Sciences, Anhalt University of Applied Sciences, Bernburg, Germany

**Keywords:** external quality assessment, proficiency testing, erythropoietin, method precision, immunoassay

## Abstract

**Introduction:** This study presents a longitudinal analysis of external quality assessment (EQA) results for erythropoietin (EPO) determinations conducted between 2017 and 2022 with a continuously increasing number of participating laboratories. The aim of this work was to evaluate participant performance and methodological aspects.

**Methods:** In each of the eleven EQA surveys, a blinded sample set of lyophilized human serum containing one sample with lower EPO concentrations (L) and one with higher EPO concentrations (H) was sent to the participating laboratories.

**Results:** A total of 1,256 measurements were included. The median (interquartile range) fraction of participants not meeting the criteria of acceptance set at 20% around the robust mean of the respective survey was 9.5% (6.1%–10.7%) (sample L) and 9.1% (5.8%–11.8%) (sample H) but lacked a clear trend in the observed period. Some surveys exhibited unusually high interlaboratory variation, suggesting interfering components in the EQA samples. Different immunological methods and reagent manufacturers also showed variability in measurement outcomes to some extent.

**Conclusion:** These findings highlight the need for continuous quality assessment in EPO measurements to ensure patient safety and identify areas for further research and investigation.

## 1 Introduction

The quantitative determination of erythropoietin (EPO) in blood is mainly performed using immunoassays. By measuring serum EPO levels, useful information can be obtained on various pathogenic changes. The resulting therapeutic algorithms can guide treatment. Chronic kidney disease, as well as systemic inflammation and malignancies, can lead to a decrease in EPO biosynthesis and, therefore, to low EPO levels in the blood (Jelkmann, 2011; Portolés et al., 2021). Higher concentrations can be measured for secondary erythrocytosis, mostly caused by hypoxemia. In addition, non-renal anemia results in a higher renal EPO production and an exponential increase in serum levels (Artunc and Risler, 2007; Jelkmann, 2011; Bunn, 2013). In combination with other parameters, EPO also serves as a marker for possible myeloproliferative diseases (Michiels et al., 2007). Endogenous EPO levels should also be determined before injecting erythropoiesis-stimulating agents to treat, for example, myelodysplasia (Fried, 2009).

An adequate measurement quality is essential for ensuring patient safety, and a formal proof of the analytical competence to measure certain parameters—namely, accreditation—is mandatory or at least recommended in most countries (Zima, 2017). Adequate treatment and patient safety require reliable test results at a consistently high standard (Laudus et al., 2022). Clinicians and especially patients expect precise test results from diagnostic testing during treatment monitoring, regardless of the laboratory performing the tests (De La Salle et al., 2017). External quality assessment (EQA) is used to independently evaluate, continuously monitor, and compare laboratory performance, and frequent participation in EQA programs is mandatory for accredited medical laboratories (Favaloro et al., 2018; Sciacovelli et al., 2018; DIN EN ISO 15189:2023-03, 2023). It is a helpful tool for accessing the current *status quo* and can help to identify areas in need of improvement (Laudus et al., 2022). Additionally, EQA can assess the precision of the methodology used by the laboratories (Favaloro et al., 2018).

Marsden et al. emphasized the need for the establishment of an EPO EQA scheme in 2006 after they found fluctuations of 2.9–200 IU/L in a sample distribution program involving six laboratories (Marsden et al., 2006). INSTAND e.V. is an independent scientific medical society and accredited organization located in Germany supporting quality assurance in medical laboratories by performing EQA in laboratory medicine. INSTAND introduced its first worldwide EQA for EPO measurements in 2017. Since then, it has been performed twice a year, and the certificate is valid for 12 months.

In order to observe developments in the general measurement quality of medical laboratories and their methodology for EPO measurement, an established EQA scheme with a certain number of participants and EQA runs is required. This study is the first to show a longitudinal analysis of the results of the INSTAND EPO EQA from 2017 to 2022 with participating laboratories from all over the world. The study also aims to summarize the results of all runs of this EQA and to present the development of the EPO EQA since its introduction.

## 2 Materials and methods

### 2.1 EPO EQA procedure

A total of eleven surveys of the EPO EQA were performed twice per year (surveys S1/S2) between 2017 and 2022, which involved an increasing number of participants from all over the world. For each survey, every participating laboratory was asked to analyze two blinded lyophilized human serum samples containing different EPO concentrations. In this work, the sample with the lower concentration is always referred to as sample L, and the one with the higher concentration is sample H. In some cases, specimens were enriched with recombinant EPO by the sample manufacturer. Due to an unexpectedly high number of participants in 2020-S1 and 2020-S2, participants were divided into two subsets (2020-S1a and 2020-S1b and 2020-S2a and 2020-S2b), and each received a different sample set. The lyophilized EQA samples had to be reconstituted with 1 mL of distilled water for 30 min at room temperature and then analyzed like a normal patient sample.

Laboratories reported their results and information about the assay they used to INSTAND via the RV-Online platform (https://rv-online.instandev.de). Between 2017 and 2022, the EQA criteria of acceptance (CoA) for EPO were set to a 20% deviation from the robust mean calculated using Algorithm A (ISO13528:2015, 2020). Laboratories that reported measurements outside the CoA would not pass the quality assessment. The German Medical Association has not yet defined a maximum permissible relative deviation in EQA schemes for EPO. Therefore, the CoA used for the evaluation of the INSTAND EPO EQA is based on the mean value of the permissible relative deviations recommended in the guideline of the German Medical Association for EQA schemes for other quantitative parameters in clinical chemistry (Bundesärztekammer, 2022).

### 2.2 Data analysis

Microsoft Excel (Version 16.56, Microsoft Corporation, Redmond, WA, USA) was used for data management. The statistical analysis and visualization of the results were performed using R Studio (Version 4.1.1 (2021-08–10), Rstudio PBC, Boston, MA, USA). Figures were created using the R-package ggplot2 (Wickham, 2016). The whiskers in the created boxplots span 1.5 times the interquartile range (IQR) above and below the box, capturing the middle 50% of the data. The dots mark outliers, which are defined as observations that exceed 1.5 times the IQR from either edge of the box.

The mean absolute deviation (MAD) to median ratio was calculated to evaluate the interlaboratory variation. Data distribution depending on the immunological methods used by the laboratories was analyzed. Methods used by the participating laboratories were enzyme-linked immunosorbent assay (ELISA), chemiluminescence immunoassay (CLIA), or luminescent enzyme immunoassay (LEIA). Reagent manufacturers’ dependent data distributions were analyzed. The manufacturers were Beckman Coulter, Inc. (BE), Siemens (DPC-Biermann; DG), and IBL International GmbH (IB). Missing information on test method and reagent manufacturer, as well as manufacturer collectives with n < 14, were grouped as “other” due to lack of statistical validity.

Nine measurements each for sample L and sample H were excluded from the dataset due to suspected sample mix-ups or data submission errors and were not included in later calculations (Supplementary Table S2).

## 3 Results

**FIGURE 1:**
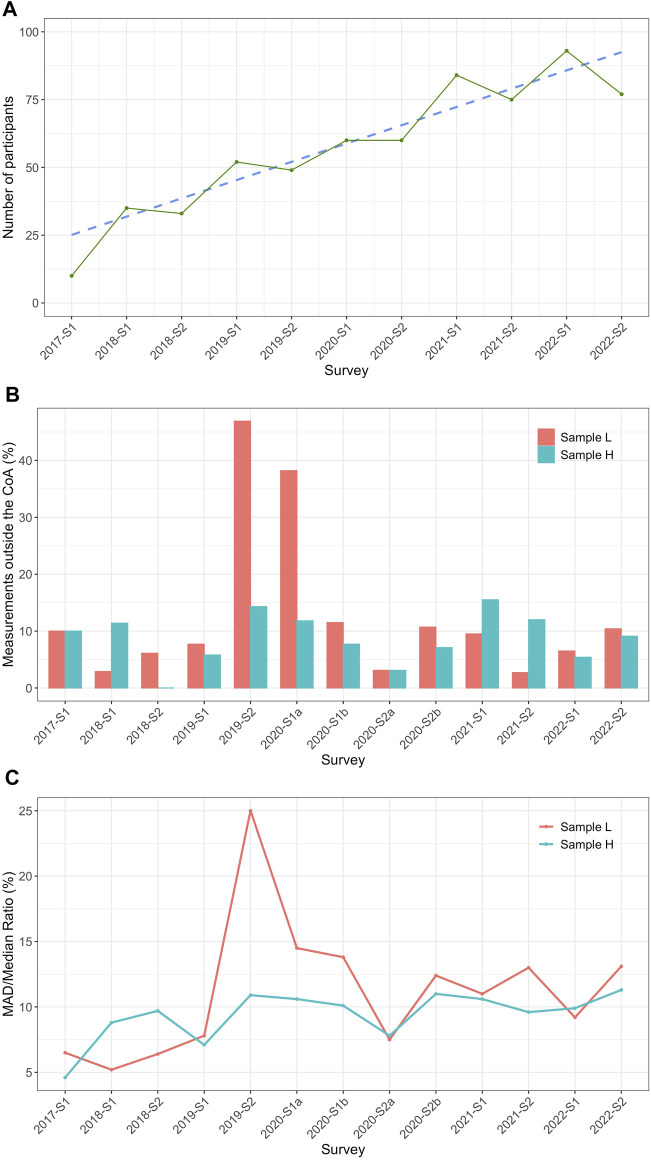
General outcome/information of the INSTAND EPO EQA from 2017 to 2022. **(A)** Number of laboratories participating between 2017 and 2022 (green) and a corresponding trend line (blue) starting with one survey (S) in 2017 (2017-S1) and continuing with two runs per year (S1/S2) until 2022. **(B)** The percentage of measurements outside the criteria of acceptance (CoA; %) calculated for each survey for sample L (red) and sample H (turquoise). The CoA was defined as ± 20% around the robust mean for the individual surveys shown. **(C)** Mean absolute deviation (MAD)/median ratio (%) for sample L (red) and sample H (turquoise) for every survey.

Overall, 1,256 measurements were evaluated. The first EQA survey conducted in 2017 had ten participating laboratories. In subsequent years, the number of participants increased to an annual average of 85 laboratories in 2022 (Figure 1; Table 1). The overall median (IQR) percentage of participants not meeting the CoA was 9.5% (6.1%–10.7%) for sample L and 9.1% (5.8%–11.8%) for sample H. Relatively high rates (46.9% and 38.2%, respectively) of measurements outside the CoA for sample L were observed for 2019-S2 and 2020-S1a (Figure 1; Table 1). The interlaboratory variation was determined by calculating the MAD/median ratio for each survey. The overall MAD/median ratio (median; IQR) was 11.0% (7.5%–13.1%) (sample L) and 9.9% (8.8%–10.6%) (sample H) but showed an unusual peak for 2019-S2 at 25.0% for sample L, which is in line with the low passing rate for this survey (Figure 1).

**TABLE 1:** Robust mean values (IU/L) calculated by Algorithm A (ISO13528:2015, 2020) and measurements outside of the criterion of acceptance (CoA) at ± 20% around the robust mean for each of the eleven surveys (S) from 2017 to 2022 for sample L (L) and sample H (H).

Survey	Robust mean (Algorithm A; IU/L)	Measurements outside the CoA
2017-S1 (n = 10)	11.0 (L)	1 (L)
	47.0 (H)	1 (H)
2018-S1 (n = 35)	10.0 (L)	1 (L)
	54.0 (H)	4 (H)
2018-S2 (n = 33)	22.0 (L)	2 (L)
	89.0 (H)	0 (H)
2019-S1 (n = 52)	13.0 (L)	4 (L)
	35.0 (H)	3 (H)
2019-S2 (n = 49)	6.6 (L)	23 (L)
	62.1 (H)	7 (H)
2020-S1a (n = 34)	6.3 (L)	13 (L)
	60.1 (H)	4 (H)
2020-S1b (n = 26)	9.9 (L)	3 (L)
	48.2 (H)	2 (H)
2020-S2a (n = 32)	12.1 (L)	1 (L)
	33.5 (H)	1 (H)
2020-S2b (n = 28)	21.7 (L)	3 (L)
	86.1 (H)	2 (H)
2021-S1 (n = 84)	10.2 (L)	8 (L)
	22.6 (H)	13 (H)
2021-S2 (n = 75)	16.2 (L)	2 (L)
	66.1 (H)	9 (H)
2022-S1 (n = 93)	17.3 (L)	6 (L)
	32.9 (H)	5 (H)
2022-S2 (n = 77)	10.2 (L)	8 (L)
	16.2 (H)	7 (H)

**FIGURE 2:**
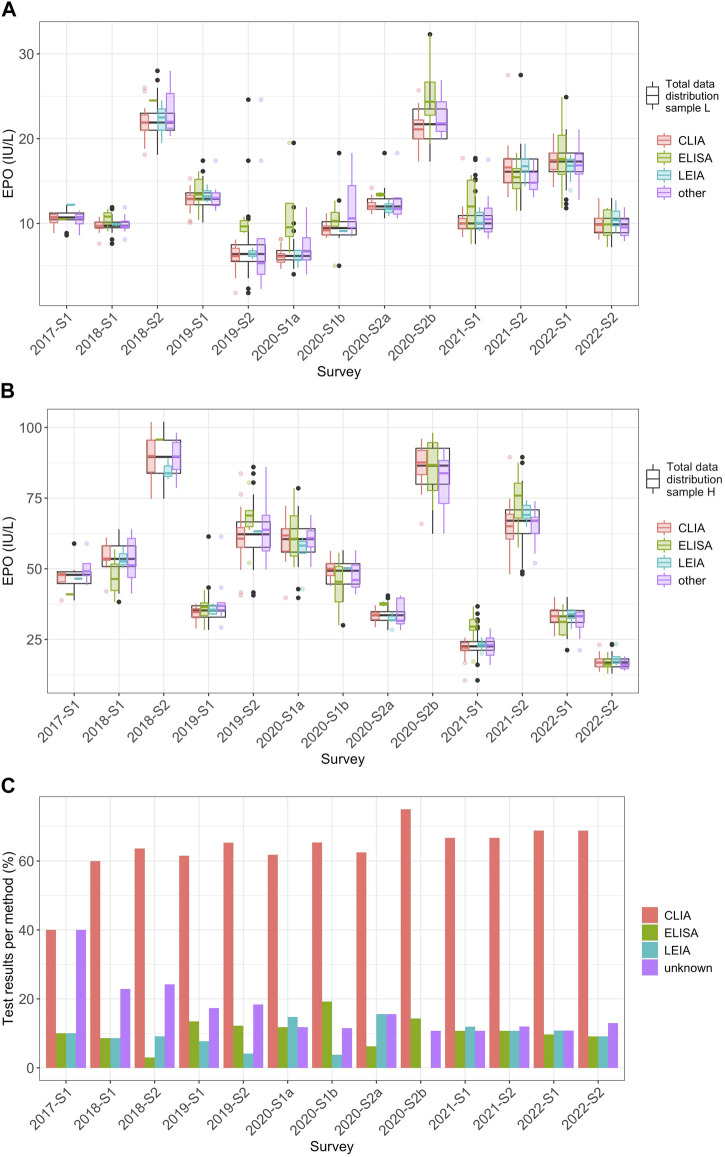
Method-dependent analysis of EQA results for EPO levels from 2017 to 2022 **(A)** Distribution of the EPO measurement results (IU/L) for the individual methods CLIA (red), ELISA (green), LEIA (turquoise), and “other” (violet) in relation to the overall distribution of all measured values in the individual surveys (black) for sample L from 2017 to 2022. In this plot, whiskers span 1.5 times the IQR above and below the box, capturing the middle 50% of the data. The red, green, turquoise, violet and black dots mark outliers, which are defined as observations that exceed 1.5 times the IQR from either edge of the box. **(B)** The same consideration used for **(A)** but for sample H. **(C)** Percentage of the frequencies for the respective measurement methods of the total of all measurements per survey per sample.

The results were also evaluated based on the immunological methods used by the laboratories (Table 2). Scatterings for the individual methods are quite low when considered in relation to the overall distribution of the data. Overall, the method-specific data distributions were mostly within the quartiles of the total data distribution. In some cases, the value distribution for ELISA shifted upwards, especially for the less concentrated samples (sample L) between 2019-S2 and 2020-S2b but also for sample H 2020-S2a and 2021-S1 (Figure 2). With 824 measurements for samples L and H combined, CLIA was the most frequently used method in every survey. LEIA had the lowest frequency, with 118 total observations. ELISA was used 132 times.

**TABLE 2:** Method-dependent and total median (interquartile range; IQR; IU/L) and respective frequencies in each survey (S) from 2017 to 2022 for sample L (L) and sample H (H).

	Median (IQR; IU/L) Frequency				
Survey	CLIA	ELISA	LEIA	other	total
2017-S1	10.8 (10.1–11.1) (L)	10.5 (-) (L)	12.2 (-) (L)	10.7 (10.0–11.1) (L)	10.7 (10.4–11.2) (L)
	47.9 (45.3–48.4) (H)	41.0 (-) (H)	46.5 (-) (H)	49.1 (47.6–51.9) (H)	47.9 (44.8–48.9) (H)
	n = 4	n = 1	n = 1	n = 4	n = 10
2018-S1	9.7 (9.4–10.1) (L)	10.8 (9.9–11.2) (L)	9.9 (9.8–10.0) (L)	9.6 (9.4–10.3) (L)	9.7 (9.5–10.1) (L)
	53.6 (53.0–58.2) (H)	46.4 (42.3–51.6) (H)	52.6 (51.5–55.3) (H)	51.2 (46.9–60.8) (H)	53.5 (50.8–58.1) (H)
	n = 21	n = 3	n = 3	n = 8	n = 35
2018-S2	21.9 (21.0–22.8) (L)	24.5 (-) (L)	22.5 (21.0–23.5) (L)	22.0 (21.2–25.3) (L)	21.9 (21.0–23.0) (L)
	89.9 (84.2–95.5) (H)	95.7 (-) (H)	83.8 (82.8–86.3) (H)	89.8 (85.1–94.7) (H)	89.6 (83.8–95.5) (H)
	n = 21	n = 1	n = 3	n = 8	n = 33
2019-S1	12.9 (12.2–13.3) (L)	13.4 (12.8–15.2) (L)	13.2 (12.7–13.8) (L)	13.0 (12.1–13.6) (L)	12.9 (12.2–13.6) (L)
	34.6 (32.7–35.9) (H)	36.4 (33.5–37.9) (H)	35.5 (34.0–37.1) (H)	36.9 (35.3–38.0) (H)	35.2 (32.9–36.9) (H)
	n = 32	n = 7	n = 4	n = 9	n = 52
2019-S2	6.1 (5.6–7.1) (L)	9.7 (9.1–10.3) (L)	6.4 (6.1–6.8) (L)	5.3 (4.0–8.2) (L)	6.4 (5.5–7.5) (L)
	60.6 (57.6–64.6) (H)	68.8 (64.9–70.6) (H)	63.2 (63.1–63.4) (H)	63.8 (56.3–68.9) (H)	62.2 (57.6–66.6) (H)
	n = 32	n = 6	n = 2	n = 9	n = 49
2020-S1a	6.1 (5.4–6.4) (L)	9.6 (8.4–12.4) (L)	5.7 (5.7–6.8) (L)	6.7 (5.7–8.3) (L)	6.2 (5.7–6.8) (L)
	61.8 (56.2–64.2) (H)	60.6 (54.5–68.8) (H)	58.2 (55.5–59.7) (H)	60.9 (57.7–63.5) (H)	60.5 (55.9–64.2) (H)
	n = 21	n = 4	n = 5	n = 4	n = 34
2020-S1b	9.2 (8.6–9.7) (L)	10.3 (9.7–11.3) (L)	9.1 (-) (L)	10.6 (9.5–14.4) (L)	9.4 (8.6–10.2) (L)
	50.0 (47.6–51.9) (H)	45.4 (38.3–50.8) (H)	50.2 (-) (H)	46.0 (43.5–51.3) (H)	49.3 (44.6–51.8) (H)
	n = 17	n = 5	n = 1	n = 3	n = 26
2020-S2a	12.0 (11.7–12.5) (L)	13.4 (13.2–13.5) (L)	11.7 (11.3–12.3) (L)	11.7 (11.1–13.0) (L)	12.0 (11.7–12.9) (L)
	33.7 (32.1–34.5) (H)	37.5 (37.1–38.0) (H)	31.8 (31.6–33.2) (H)	31.6 (30.5–39.6) (H)	33.5 (31.8–34.8) (H)
	n = 20	n = 2	n = 5	n = 5	n = 32
2020-S2b	21.1 (20.0–22.2) (L)	24.4 (22.8–26.7) (L)	-	21.8 (20.9–24.4) (L)	21.7 (20.0–23.5) (L)
	87.6 (83.3–91.9) (H)	86.8 (77.7–94.7) (H)	-	83.8 (73.1–88.3) (H)	86.5 (79.9–92.6) (H)
	n = 21	n = 4	n = 0	n = 3	n = 28
2021-S1	10.0 (9.4–10.6) (L)	12.0 (9.4–15.1) (L)	10.1 (9.5–11.3) (L)	10.5 (9.0–11.8) (L)	10.0 (9.4–10.9) (L)
	22.0 (21.0–23.8) (H)	29.5 (28.3–32.0) (H)	23.1 (22.4–23.9) (H)	23.0 (19.4–25.5) (H)	22.6 (21.1–24.2) (H)
	n = 56	n = 9	n = 10	n = 9	n = 84
2021-S2	16.6 (14.9–17.7) (L)	15.4 (14.1–16.5) (L)	16.8 (16.0–17.7) (L)	14.8 (14.0–16.0) (L)	16.1 (14.8–17.6) (L)
	65.0 (60.5–69.3) (H)	75.9 (68.0–80.3) (H)	69.0 (67.9–72.4) (H)	66.9 (62.5–68.2) (H)	67.0 (62.5–70.8) (H)
	n = 50	n = 8	n = 8	n = 9	n = 75
2022-S1	17.4 (16.3–18.3) (L)	17.6 (15.8–20.4) (L)	16.8 (16.2–17.6) (L)	16.9 (15.8–18.2) (L)	17.3 (16.1–18.3) (L)
	33.2 (31.3–35.5) (H)	31.2 (26.6–33.3) (H)	34.0 (32.5–35.4) (H)	33.3 (29.3–34.6) (H)	33.2 (31.0–35.2) (H)
	n = 64	n = 9	n = 10	n = 10	n = 93
2022-S2	9.8 (9.0–10.6) (L)	9.9 (8.6–11.6) (L)	10.4 (9.7–11.4) (L)	9.5 (8.6–10.4) (L)	9.9 (8.9–10.6) (L)
	16.8 (15.3–18.1) (H)	16.1 (15.2–18.0) (H)	18.0 (16.6–18.9) (H)	16.0 (14.6–17.4) (H)	16.8 (15.3–18.1) (H)
	n = 53	n = 7	n = 7	n = 10	n = 77

**FIGURE 3:**
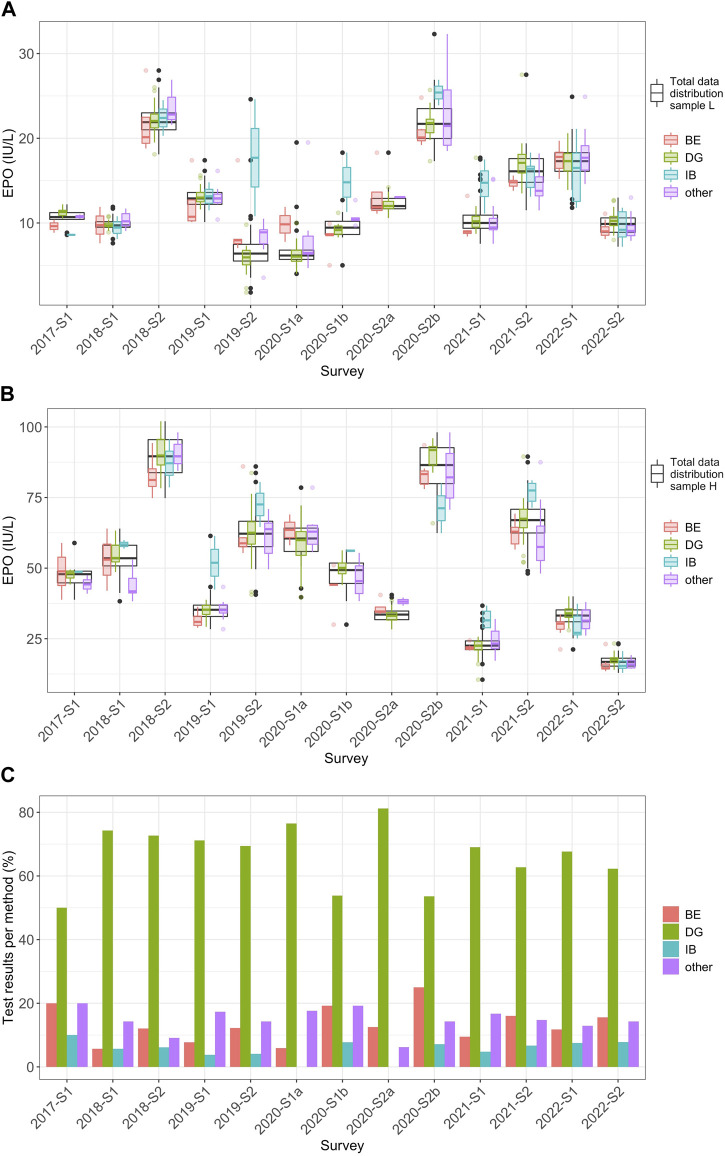
Manufacturer-dependent analysis of EQA results for EPO levels from 2017 to 2022. **(A)** Distribution of the EPO measurement results (IU/L) for the individual reagent manufacturers BE (red), DG (green), IB (turquoise), and “other” (violet) in relation to the overall distribution of all measured values in the individual surveys (black) for sample L from 2017 to 2022. In this plot, whiskers span 1.5 times the IQR above and below the box, capturing the middle 50% of the data. The red, green, turquoise, violet and black dots mark outliers, which are defined as observations that exceed 1.5 times the IQR from either edge of the box. **(B)** The same consideration used in **(A)** but for sample H. **(C)** Percentage of the frequencies for the respective manufacturers of the total of all measurements per survey per sample.

Regarding the reagent manufacturer-dependent data analysis, the most frequently used manufacturer was DG, with 846 measurements for sample L and sample H combined (Table 3). Manufacturer BE was used 158 times. IB was used the least (n = 70). IB showed a tendency for higher values, and upward shifts could be observed in some surveys, especially for sample L between 2019-S2 and 2021-S1, but also for sample H in the 2019 and 2021 surveys (Figure 3). In some cases, BE tended towards values in the lower range of the overall distribution and, in some cases, even outside the lower quartile. One shift outside the upper quartile could also be seen for sample L in 2020-S1a. Manufacturer DG mostly showed values in the mid-range of the overall data.

**TABLE 3:** Manufacturer-dependent and total median interquartile range (IQR; IU/L) and respective frequencies in each survey (S) from 2017 to 2022 for sample L (L) and sample H (H).

	Median ± IQR (IU/L) Frequency				
Survey	BE	DG	IB	other	total
2017-S1	9.6 (9.2–10.0) (L)	11.3 (10.9–11.5) (L)	8.6 (-) (L)	10.8 (10.6–10.9) (L)	10.7 (10.4–11.2) (L)
	48.8 (43.8–53.9) (H)	48.2 (46.5–49.0) (H)	48.7 (-) (H)	44.2 (42.6–45.9) (H)	47.9 (44.8–48.9) (H)
	n = 2	n = 5	n = 1	n = 2	n = 10
2018-S1	9.8 (8.7–10.8) (L)	9.7 (9.6–10.0) (L)	9.4 (8.8–10.1) (L)	10.2 (9.7–11.1) (L)	9.7 (9.5–10.1) (L)
	53.0 (47.5–58.5) (H)	53.6 (52.2–58.2) (H)	58.4 (57.6–59.1) (H)	41.8 (41.2–46.4) (H)	53.5 (50.8–58.1) (H)
	n = 2	n = 26	n = 2	n = 5	n = 35
2018-S2	20.1 (19.4–22.5) (L)	22.0 (21.2–22.9) (L)	22.4 (21.4–23.4) (L)	22.8 (22.2–24.9) (L)	21.9 (21.0–23.0) (L)
	81.2 (78.9–85.2) (H)	89.9 (86.5–95.6) (H)	87.2 (82.9–91.4) (H)	89.6 (87.0–93.8) (H)	89.6 (83.8–95.5) (H)
	n = 4	n = 24	n = 2	n = 3	n = 33
2019-S1	10.7 (10.2–12.7) (L)	13.0 (12.5–13.6) (L)	13.2 (12.3–14.0) (L)	12.9 (12.4–13.4) (L)	12.9 (12.2–13.6) (L)
	30.9 (29.7–33.0) (H)	35.3 (33.6–36.8) (H)	51.9 (47.1–56.6) (H)	35.1 (34.1–37.1) (H)	35.2 (32.9–36.9) (H)
	n = 4	n = 37	n = 2	n = 9	n = 52
2019-S2	8.0 (7.6–8.1) (L)	5.9 (5.1–6.8) (L)	17.7 (14.3–21.2) (L)	8.9 (7.6–9.2) (L)	6.4 (5.5–7.5) (L)
	58.8 (57.5–60.7) (H)	62.5 (58.5–66.4) (H)	72.6 (68.6–76.5) (H)	63.8 (55.2–65.9) (H)	62.2 (57.6–66.6) (H)
	n = 6	n = 34	n = 2	n = 7	n = 49
2020-S1a	9.8 (8.8–10.9) (L)	6.0 (5.5–6.8) (L)	-	6.5 (6.3–8.4) (L)	6.2 (5.7–6.8) (L)
	63.5 (60.8–66.3) (H)	59.9 (54.5–62.9) (H)	-	62.9 (58.4–65.2) (H)	60.5 (55.9–64.2) (H)
	n = 2	n = 26	-	n = 6	n = 34
2020-S1b	8.6 (8.5–8.7) (L)	9.2 (8.7–9.6) (L)	14.8 (13.1–16.6) (L)	10.4 (10.3–10.6) (L)	9.4 (8.6–10.2) (L)
	44.1 (43.9–44.3) (H)	50.1 (48.0–51.8) (H)	56.2 (55.9–56.4) (H)	45.4 (41.0–50.8) (H)	49.3 (44.6–51.8) (H)
	n = 5	n = 14	n = 2	n = 5	n = 26
2020-S2a	11.8 (11.5–13.6) (L)	12.0 (11.7–12.5) (L)	-	13.1 (13.0–13.1) (L)	12.0 (11.7–12.9) (L)
	34.6 (34.3–36.2) (H)	33.1 (31.6–34.2) (H)	-	38.2 (37.4–38.9) (H)	33.5 (31.8–34.8) (H)
	n = 4	n = 26	n = 0	n = 2	n = 32
2020-S2b	20.1 (19.7–21.0) (L)	21.8 (20.6–22.2) (L)	25.4 (24.6–26.1) (L)	21.4 (19.2–25.7) (L)	21.7 (20.0–23.5) (L)
	83.3 (79.7–84.4) (H)	91.8 (86.5–93.0) (H)	71.2 (66.8–75.6) (H)	82.2 (74.8–90.6) (H)	86.5 (79.9–92.6) (H)
	n = 7	n = 15	n = 2	n = 4	n = 28
2021-S1	9.0 (8.8–9.1) (L)	10.2 (9.5–10.8) (L)	14.8 (13.1–16.1) (L)	9.5 (9.2–10.5) (L)	10.0 (9.4–10.9) (L)
	21.6 (21.2–22.1) (H)	22.5 (21.0–24.0) (H)	31.5 (28.9–34.8) (H)	23.3 (21.6–27.7) (H)	22.6 (21.1–24.2) (H)
	n = 8	n = 58	n = 4	n = 14	n = 84
2021-S2	14.8 (14.4–15.0) (L)	17.1 (15.9–18.1) (L)	16.4 (14.2–16.7) (L)	13.8 (13.2–15.4) (L)	16.1 (14.8–17.6) (L)
	62.9 (58.6–64.4) (H)	67.5 (64.3–70.8) (H)	77.5 (73.7–80.0) (H)	57.5 (52.7–64.9) (H)	67.0 (62.5–70.8) (H)
	n = 12	n = 47	n = 5	n = 11	n = 75
2022-S1	17.8 (16.4–18.4) (L)	17.3 (16.1–18.2) (L)	16.5 (12.6–18.2) (L)	17.7 (16.2–19.1) (L)	17.3 (16.1–18.3) (L)
	30.3 (28.4–31.0) (H)	34.0 (32.5–35.5) (H)	27.1 (26.4–33.0) (H)	31.4 (28.5–35.1) (H)	33.2 (31.0–35.2) (H)
	n = 11	n = 63	n = 7	n = 12	n = 93
2022-S2	9.0 (8.6–9.6) (L)	10.2 (9.7–10.8) (L)	9.2 (8.4–11.3) (L)	9.1 (8.5–9.7) (L)	9.9 (8.9–10.6) (L)
	14.6 (14.3–16.0) (H)	17.5 (16.5–18.2) (H)	15.4 (14.5–17.1) (H)	15.9 (14.7–17.4) (H)	16.8 (15.3–18.1) (H)
	n = 12	n = 48	n = 6	n = 11	n = 77

## 4 Discussion

This study summarizes quantitative EQA results for EPO determination conducted between 2017 and 2022. The MAD/median ratio was below 15% in almost every case. Survey 2019-S2 showed higher values at 25.0% for sample L. Also, some immunological methods and reagent manufacturers showed variability in measurement outcomes to some extent. These findings should also be placed in relation to their clinical relevance. EPO determination is mainly a diagnosis of exclusion to identify, for example, chronic kidney disease as the cause of anemia. Therefore, the focus is on the concentration of EPO in relation to other anemia markers rather than on the exact prevailing EPO concentration. Low EPO concentrations in the blood, in combination with hemoglobin concentrations below 13.0 g/dL (adult males) and 12.0 g/dL (non-menstruating females), may indicate a renal cause (Lankhorst and Wish, 2010). Non-renal anemia usually results in increased EPO levels, and, in severe cases, an increase of up to 1000-fold can be reached (Artunc and Risler, 2007; Higgs et al., 2015). Hence, measurement deviations may be, to a small extent, clinically less critical if the EPO value is considered in relation to the relevant biomarkers. Nevertheless, clinical laboratories should always strive for the highest measurement precision so that patient safety, as the highest priority, is never compromised. To this date, further investigation is needed to get clear statements on quality specifications for EPO measurement variation.

Scattering in the EPO levels of the investigated immunological methods and reagent manufacturers could be observed in some cases. Immunoassays have an analytical error rate of 0.4%–4% (Ismail, 2017). This can be attributed to exogenous factors such as variability in sample pipetting and other handling errors or systematic exogenous error sources such as calibration errors (Sturgeon and Viljoen, 2011). Furthermore, interfering factors, such as the reagents used, have been known to affect measurement outcomes (Alhajj and Farhana, 2022). There also may be excessive non-specific binding of the antibody or antigen in the assay performed (Gan and Patel, 2013). It is known that the imprecision of EPO quantification immunoassays depends on the concentration (Marsden, 2006). Especially for the reagent manufacturer IB, scatter could be observed at median sample concentrations of 10 IU/L or less. This manufacturer was only used in combination with the ELISA and “other” method collective. The concentration range of the calibration curve is 10.7–469 IU/L of the commercially available ELISA kit from this manufacturer, according to the manufacturer’s website (IBL International GmbH, 2023). Thus, the EPO concentration in the samples might have been too close to the detection limit of the assay. However, due to the comparably small number of IB applications, more measurements would be needed to corroborate this assumption. Compared to IB, the lowest limit of detection for the manufacturer DG device Immulite 2000 was found to be 0.16 IU/L, with the manufacturer’s recommended detection limit being 0.24 IU/L (Benson et al., 2000). The lowest limit of detection for the DG device Advia Centaur Systems is given at 0.75 IU/L (Siemens Healthcare Diagnostics Inc, 2019). The dynamic range of the BE family of Access Immunoassay Systems EPO assays could be determined at 0.6–750 IU/L (Retka et al., 2005; Beckman Coulter, Inc., 2023). Marsden et al. compared different EPO ELISA test kits with radioimmunoassay as a reference test. One kit from the manufacturer IB was also included in that comparison and showed a slight positive bias compared to the reference method. Even though Marsden et al. was conducted in 1999, and no radioimmunoassay was used in the present study, these results are in line with some observed upward shifts for this manufacturer (Marsden et al., 1999).

In some cases, slight fluctuations were also observed for BE. Owen and Roberts compared the test performance of the Access 2 device of this manufacturer with the Immulite 2000 device by the manufacturer DG and obtained comparably good results with both manufacturers (Owen and Roberts, 2011). As the sample sizes for both manufacturers were the same in the study mentioned (n = 101) compared to the extremely varying frequencies of use in this EQA, the results obtained here do not yet indicate a clear difference in the measurement range of the two methods. Owen and Roberts also compared the two manufacturers DG and BE in terms of cross-reactivity with recombinant EPO preparations and found that both differed considerably in the measurement results of samples spiked with Epoetin alfa and Darbepoetin alfa, as the values for BE were in a much higher range—109 IU/L higher and 242 IU/L higher than DG, respectively (Owen and Roberts, 2011). Because the samples used for these EQA surveys were sometimes spiked, differences in cross-reactivity with recombinant EPO as the cause of variability cannot be safely excluded.

The manufacturer DG was used most frequently by the EQA participants in this work. A study by Abellan et al. from 2004 compares the Immulite 2000 system from DG, which is based on CLIA, with an ELISA kit by a different manufacturer that was not used by any participant in the present study. The DG device showed better intra-laboratory precision and a lower variation in the interlaboratory comparison. Both immunoassay methods correlated well, although ELISA tended to show lower values (Abellan et al., 2004). In the methodological comparison of the present study, some cases were observed in which ELISA tended to show higher values than CLIA and LEIA, which contrasts with the tendency observed in the mentioned article.

Because there is not yet any reference method for quantitative EPO determination, no valid statement can be made as to which method or which manufacturer offers the highest precision. External quality controls are, therefore, even more important when comparing the measuring ranges of the laboratories and the methodology. Methodological comparisons require representative sample sizes, which are partially not yet given due to the low frequency of use in some cases. Because the number of participants in the EPO EQA has been increasing, more specific comparisons might be made in future studies.

It should also be noted that the standards used for the IBL-ELISA were calibrated against the first international erythropoietin standard (87/684) (IBL International GmbH, 2022 National Institute for Biological Standards and Control, 2008). The calibrator of the Immulite 2000 by manufacturer BE and the devices used in this study from manufacturer DG are traceable to the second international erythropoietin standard (67/343) (Owen and Roberts, 2011; Beckman Coulter, Inc., 2020). The second international standard is derived from urine but is used to calibrate detection in human serum or plasma (National Institute for Biological Standards and Control, 2013). It remains questionable whether accurate results can be obtained in blood if the calibrators of the assays are traceable to a standard from a completely different matrix. The Siemens Advia Centaur device from manufacturer BE, which was used by some participants in this study, is traceable to the second international standard and the third international erythropoietin standard (11/170), which is mainly based on a recombinant EPO preparation (National Institute for Biological Standards and Control, 2012; Siemens Healthcare Diagnostics Inc, 2019).

EQAs may not be passed for different reasons, most of which can be attributed to human error, such as sample mix-ups or errors during the reconstitution process. Li et al. found that potential reasons for not passing EQAs can, for example, be due to errors in the management of the measurement results, such as transcription errors or reporting of incorrect units, which were also noticed in this work. However, technical errors, such as calibration problems, were described as the main reason (Li et al., 2019). To successfully complete the EQA, it is important that participants follow the details of the test scheme and apply good laboratory practices, like checking the methods for quality and ensuring that the staff is adequately trained (Edson et al., 2007). Two surveys (2019-S2 and 2020-S1a) did stand out with a particularly high failure rate and high interlaboratory variation for sample L. The same batch of sample sets was used in these two surveys. This suggests that there might be interfering components in this batch for sample L. This may be due to unusually high concentrations of regular serum components prevailing in the sample, leading to falsely high or falsely low results (Sequeira, 2019). Insufficient commutability of the sample may also have negatively impacted the test performance. It is often not possible to use authentic clinical samples in the context of proficiency testing. However, artificially generated samples do not always mirror the patient samples that are routinely examined in laboratories (Laudus et al., 2022). In the EQA surveys performed, samples were sent to participants in lyophilized form. The samples used in 2019-S2 and 2020-S1a were not spiked with recombinant EPO, but other samples used in this study were. Both sample preparation and sublimation have been described as possible influencing factors (Vesper et al., 2007; Miller et al., 2011). As mentioned above, there can also be differences in cross-reactivity with recombinant EPO preparations depending on the assay manufacturer (Owen and Roberts, 2011).

The study had the following limitations: The exact isoform of recombinant EPO spiked into some of the samples is unknown. This makes it difficult to draw conclusions about any possible cross-reactivity in the samples. It should also be reiterated here that commutability studies of the EQA samples have not yet been carried out, so a possible influence of the sample preparation on EPO detection is not known. Whether the test performance is affected by the sample itself should be evaluated. As mentioned above, there is no validated reference method for quantitative EPO detection. Accordingly, no analytical target value can be determined for the evaluation of the EQA, and the robust mean value must be used as the target value for evaluation, which is a common practice. The most represented method or manufacturer also has the strongest influence on the overall mean. Because the true value is unknown, this can lead to biases in the evaluation to an unknown extent (Kristensen and Meijer, 2017). Furthermore, it is not possible to include the exact specifications given by the manufacturer for each method at any given time, as the corresponding reagent kits and batches are not known. The EQA is also intended to provide an overall picture of the analyses rather than comparing individual kits and batches.

However, the results presented in this study are of importance despite the limitations mentioned, as this is the first longitudinal evaluation of EPO EQA data to date. Medical laboratories should always aim to keep their measurement quality at the highest standard, and this work can be used to reflect on the institution’s methodology and to see how their detection method or the assay manufacturer used performs in relation to others.

## 5 Conclusion

This work shows that variations in laboratory results and in methodological terms for quantitative EPO determination do persist to some degree, and knowledge about sources of errors is vital in order to optimize measurement quality and thus ensure patient safety. However, in terms of clinical relevance, small deviations might be considered less critical for the diagnostic assessment and the resulting therapeutic consequence in patients because, in anemia diagnostics, the level of EPO in combination with other relevant biomarkers is of decisive importance. Thresholds for maximum acceptable variation in EPO measurement quality and their clinical consequences should be further investigated in the future.

## Data Availability

The original contributions presented in the study are included in the article/Supplementary Material; further inquiries can be directed to the corresponding author.

## Supplementary material

Supplementary Table 1: Overview of the collectives considered in the data analysis and corresponding information on the reagent manufacturers and devices used.

Supplementary Table 2: Raw data.
