# Supplementary material for: Longitudinal evaluation of laboratory results and method precision in worldwide erythropoietin external quality assessments
Source: Front Mol Biosci. 2024 Jun 21;11:1390079. doi: 10.3389/fmolb.2024.1390079 (PMC11224661; doi:10.3389/fmolb.2024.1390079)
Supplement: Supplementary file 2 [file Table1.pdf]

Supplementary Table 1. Overview of the collectives considered in the data analysis and corresponding information on the reagent manufacturers and devices used

Method	Reagent Manufacturer	Device
other/ not specified	Beckman Coulter (BE)	not specified
	Siemens (DPC Biermann; DG)	Siemens Advia Centaur XP
		Siemens (DPC Biermann) Immulite 2000
		Siemens: other
	IBL (IB)	not specified
	other/not specified	Beckman Unicel DX System
		Siemens (DPC Biermann) Immulite 2000
		not specified
CLIA	Beckman Coulter (BE)	Beckman Unicel DX System
		Beckman Access
	Siemens (DPC Biermann; DG)	Siemens Advia Centaur XP
		Siemens (DPC Biermann) Immulite 2000
		Siemens: other
		not specified
	other/not specified	Siemens Advia Centaur XP
		Siemens (DPC Biermann) Immulite 2000
		Siemens: other
		not specified
ELISA	IBL (IB)	Siemens: other
		not specified
	Siemens (DPC Biermann; DG)	not specified
	Beckman Coulter (BE)	not specified
	not specified	not specified
LEIA	Siemens (DPC Biermann; DG)	Siemens (DPC Biermann) Immulite 2000
